# Learning Behavior Evaluation Model and Teaching Strategy Innovation by Social Media Network Following Learning Psychology

**DOI:** 10.3389/fpsyg.2022.843428

**Published:** 2022-07-22

**Authors:** Lijuan Yuan, Hongming Li, Shiman Fu, Zizai Zhang

**Affiliations:** ^1^College of Journalism and Communications, Zhoukou Normal University, Zhoukou, China; ^2^Southampton Education School, University of Southampton, Southampton, United Kingdom; ^3^Hangzhou Preschool Teachers College, Zhejiang Normal University, Hangzhou, China

**Keywords:** online learning, deep learning, encoder, network embedding model, teaching strategies

## Abstract

With the development of various network technologies and the spread of coronavirus disease 2019, many online learning platforms have been built. However, some of them may negatively impact student learning outcomes. Therefore, this study aims to improve the online learning effect of students by comprehensively evaluating their learning behavior by using deep learning algorithms. On this basis, new teaching strategies are proposed. According to the structured deep network embedding model, a network representation learning algorithm is proposed with the help of auto-encoders under deep learning. This study elaborates the concept and structure of the encoder model and tests its performance. After the node labels and dataset are trained, the applicable parameter λ_2_ of the model is 0.3. During the teaching process, the model’s reliability in distinguishing users is examined. Therefore, this model can be applied to network teaching, is an innovative teaching strategy, and provides a theoretical basis for improving teaching methods.

## Introduction

With the rapid development of network technology, social media networks play an increasingly important role in people’s daily life, work, and entertainment and become indispensable (Zahara et al., 2021). Social media networks can meet people’s communicative needs without meeting the person face to face. The scale of social media networks and the size of users continue to expand every year. These changes bring unprecedented challenges to the use of network media (Hoi et al., 2020). In particular, the scale of social media networks expands sharply and reaches an unprecedented level due to coronavirus disease 2019 (COVID-19) (Adnan and Anwar, 2020). For example, network platforms such as nails and the superstar learning network platform have become the most frequently used online teaching platforms (Kent and Rechavi, 2020).

There are many forms of networks in the real world, such as citation networks, social networks, etc. These networks describe a certain relationship between each object and other objects and have important practical significance for data analysis and practical applications. Traditional research methods usually describe a network abstractly as a discrete adjacency matrix. Among them, each vertex of the matrix represents an object in the network. Each element in the matrix represents the adjacency relationship between objects (Sun et al., 2021). However, this representation not only needs to calculate the relationship between each pair of data, but also is difficult to describe the more complex structural relationship between the data, such as the path relationship between nodes and so on. This makes this class of methods problematic when dealing with large-scale network tasks. On the one hand, the adjacency matrix needs to calculate the adjacency relationship between each pair of samples, which will greatly increase the computational cost (Liang and Markchom, 2022). On the other hand, this simple adjacency relationship representation is difficult to describe more complex relationships between data, which is difficult to accurately describe the information of network structure. Therefore, how to construct a graph that can accurately describe the structural relationship of network vertices and reduce the high computational cost has attracted extensive attention. Network representation learning algorithms embed the graph into a vector space, which preserves the structure and inherent properties of the graph (Chen et al., 2022). Network representation learning is mainly used to learn vector representations of real data in low-dimensional latent spaces. Additionally, the original topology structure of the real network, including the information on vertices and edges, is maintained during the learning process. Thereafter, traditional machine learning algorithms are applied to the learned low-dimensional space for subsequent network analysis tasks. This representation method can not only describe the complex network structure more accurately but also transform the analysis task of the original network into the analysis of the potential low-dimensional network space, which is beneficial to saving the computational cost (Guo et al., 2021; Nguyen et al., 2021).

How to provide effective teaching strategies for students is still an urgent problem to be solved. Since many online platforms only set up the live broadcast function to provide students with the real-time viewing process of online teaching, the functions for students’ after-class course playback, after-class homework, and guidance are relatively lacking. Therefore, the auto-encoder (AE) in deep learning (DL) is used to establish a model similar to the neural network, and a network representation learning technology is proposed. According to the concept of DL, the establishment process of the model is explained, and the accuracy of node classification of the model is analyzed. Then, the classification ability for normal users and unrelated users is analyzed to identify the applicability of the model. In order to gradually narrow the educational gap among regions, urban and rural areas, and schools, Xingnan Primary School has determined the working idea of “taking platform construction as the basis, focusing on innovative application, leading demonstration as the starting point, deeply integrating information technology and education, and promoting joint construction and sharing of high-quality educational resources.” Schools should constantly strengthen the construction of the platform, innovate network teaching and research methods, and improve the effect of network classroom teaching. The innovation is to use DL to classify normal users and unrelated users.

## Literature Review

Social media network platforms play different roles in people’s lives. For example, Facebook is for entertainment as a WeiBo platform, Zhihu, and Twitter are forums for people to express their views, and nails are used as learning platforms. In recent years, many research experts have been exploring the relationship between teaching quality and teaching methods. Wu and Chen (2021) introduced a method of designing courses in the case of limited prior knowledge for NPD courses. In their study, potential semantic analysis is used to extract the main research topics from journals, which provides a new basis for the innovation and management of curriculum development (Wu and Chen, 2021), Wu et al. (2017) studied the relationship between the entrepreneurial knowledge system and traditional teaching methods and gave some suggestions on the future theoretical direction of the entrepreneurial knowledge system, improving the acceptance of entrepreneurship and promoting economic development. There are also some studies that focus on the psychological factors in online learning. Rogoza et al. (2018) analyzed the influence of fragile psychology, narcissism, admiration, and competition on the teaching quality in the learning process. The results show that fragile narcissism and admiration are contradictory to shyness and loneliness, and liking competitions reflects that the person has low empathy. Similarly, Wang et al. (2019a) explored the relationship between the driving forces behind the entrepreneurial intention of online teaching and practice and revealed that psychological states had a certain impact on entrepreneurship education and practical education. In addition, many experts discussed the learning effects of different online teaching methods on students’ academic performance. For example, Lai et al. (2019) conducted a study on whether learning media affect students’ academic performance and participation and found that the learners who participated in the teaching activities actively had better academic achievements. Some innovative suggestions for students’ teaching strategies through social media networks will be proposed.

Early research on network representation learning was mainly based on factorization methods. This type of method mainly adopts the idea of graph embedding to explore the manifold structure of the data (Chen et al., 2021). On the premise that the input data are independent and identically distributed, the graph embedding method believes that the data has a manifold structure, so the similarity between the data is calculated first. A graph describing the relationship of the network structure is constructed, and then the relationship graph is embedded in the low-dimensional subspaces (Fu et al., 2021). The local linear embedding method describes the reconstruction relationship between local neighbor nodes with a graph, and maintains this local reconstruction relationship in a low-dimensional space. The Laplacian feature mapping method enables the latent low-dimensional space to still maintain the data similarity relationship in the original space, which is used to preserve the nonlinear structure of the data (Gao et al., 2021). However, when such methods are applied to large-scale networks, the computational complexity is greatly affected by the number of vertices, and the scalability is limited. Shang et al. (2021) proposed a multilayer network representation learning method (NRLM) for drug-target interaction prediction. The method can integrate useful information from different networks, reduce noise in multilayer networks, and learn feature vectors for drugs and targets. Eigenvectors of drugs and targets are put into the drug-target space to predict potential drug-target interactions. The multilayer NRLM and prediction accuracy are improved by adding parameter regularization constraints. Multi-label learning has received extensive attention for its applicability to machine learning problems. In recent years, in order to improve the efficiency of multi-label classification, scholars have proposed several methods based on extreme value learning machine (ELM) or Radial Basis Function (RBF) neural network. Most of the existing multi-label learning algorithms mainly focus on the information of the feature space. Rezaei-Ravari et al. (2021) used two neural network structures, namely multi-label RBF and multi-label multilayer ELM. Then, a regularized multi-label learning method based on feature manifold learning and a regularized multi-label learning method based on two-manifold learning are established to train the two network structures. Experiments show that two-manifold learning is used as a training method for neural classifiers, and using some cutting-edge multi-label techniques can significantly improve classification efficiency.

In short, the NRLM based on matrix decomposition can reconstruct the network, but it is prone to overfitting, and the overall performance is not satisfactory. Although the network representation learning algorithms based on natural language models can use the network structure to learn network representations, they are all shallow models. This also means that it is difficult for them to learn the deeper and more complex features of the network structure. DL has developed rapidly in recent years and has made important progress in many fields. Its essence is to perform deep abstraction on data features and learn the mapping function from high-dimensional data to low-dimensional features. Network representation learning can also be regarded as the process of converting the representation of nodes from the high-dimensional space of the original network to a low-dimensional vector space. The essential problem is to learn the mapping function between two vector spaces. Therefore, AE in DL are used to build models like neural networks, and a network representation learning technique is proposed. The purpose is to study how the relevant technology is used.

## Materials and Methods

### Auto-Encoder Under Deep Learning

**FIGURE 1:**
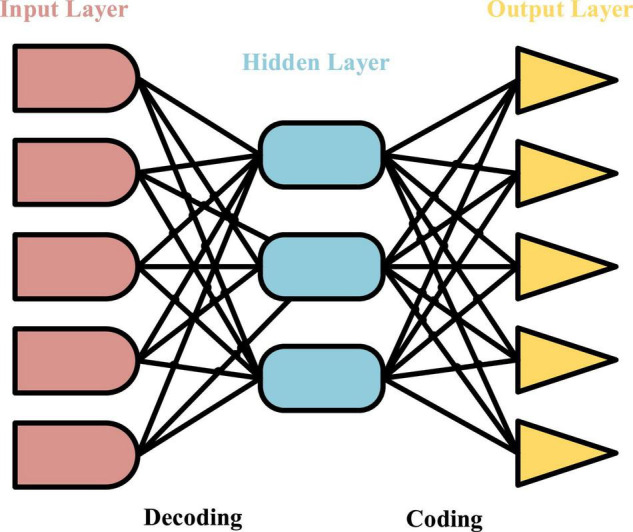
Structure of auto-encoder.

The essence of the network representation learning proposed is to transform each node in the high-dimensional space into a vector in a relatively low-dimensional space, that is, the mapping function between every two vectors is learned and applied. AE is a model similar to a neural network model (Wang and Cha, 2021), and its structure is shown in Figure 1. It regards the hidden layer in the neural network as one of the encoders and decoders to process the data imported into the network model, which need to be encoded and decoded. When the data reach the output layer, it should be confirmed that the output data are consistent with the input prototype data. In other words, the hidden layer is responsible for ensuring that the processed data are consistent with the original data because it can identify the characteristics of the input data and retain these characteristics and features. AE is a neural network model to realize that the data are input. Finding the main factors that represent the prototype data is an important work to identify the characteristics of the input data. A variety of encoders are combined to form a stack auto-encoder (SAE) (Wang et al., 2020).

The performance parameters of the model can be obtained after the model is trained. If a training set has m training samples *x*1,*x*2,…,*xn*, they must meet the condition of x∈iRN.

The operation process is that *x^i^* passes through weight matrix W∈1RK⁢x⁢N in the input layer and the hidden layer, where K is the number of hidden nodes. Then, the data are encoded by bias vector b_1_ ∈ *R^K^*, and the characteristics of the K dimension in the hidden layer are obtained. The equation is as follows:


(1) $$f\,\left(x_{i}\right)=\sigma \,\left(w_{1}\,x_{i}+b_{1}\right)$$

σ(*ldot*) is a nonlinear activation function.

When the data are decoded, the weight matrix W2 ∈ *R^NxK^* and offset vector b2 ∈ *R^N^* in the hidden layer and output layer can be used to construct the vector with dimensions N and K from the feature data. The equation is:


(2) $$g\,\left(x_{i}\right)=\sigma \,\left(W_{2}*f\,\left(x_{i}\right)+b_{2}\right)$$

The whole training process is to constantly adjust weight matrices W_1_ and W_2_ and bias vectors b_1_ and b_2_, and the objective function is minimized by:


(3) $$L_{S\,A\,E}=\frac{1}{2\,m}\,\sum_{i=1}^{m}{||g\,\left(f\,\left(x_{i}\right)\right)-x_{i}||}^{2}$$

$$+\frac{\tau }{2}\,{\left|+\left|W_{1}\right|\right|}^{2}+\frac{\tau }{2}\,{||W_{2}||}^{2}$$

||…||^2^ represents the square of norm L2, and τ is a hyperparameter.

The structure of contractive auto-encoder (CAE) (Aamir et al., 2021) is similar to that of AE. The difference is that a new penalty term for error reconstruction is added, and it is viewed as the activation function for input data. Its main function is to capture the changes in training data samples. The advantage of CAE is that the extracted features are robust to the subtle changes in the training samples. The activation function of the encoder is used to improve the robustness of the data, which is realized by:


(4) $${||J\,\left(x\right)||}_{F}^{2}=\sum_{i\,j}{\left(\frac{\partial f_{i}\,\left(x_{i}\right)}{\partial x_{i}}\right)}^{2}$$

In the above equation, *J*(*x*) represents the Jacobian matrix (Yan et al., 2018), j represents the total number of hidden layers, so the total number of objective functions of the CAE is as follows:


(5) $$L_{C\,A\,E}=\frac{1}{2\,m}\,\sum_{i=1}^{m}{||g\,\left(f\,\left(x_{i}\right)\right)-x_{i}||}^{2}+\tau \,{||J\,\left(x\right)||}_{F}^{2}$$

In Equation 5, the first half on the right side of the equal sign is the minimum reconstruction error, and the second half is to make the model network unchanged locally. The value of the partial derivative should be minimized in the calculation. If the partial derivative is 0, the model is robust to the vibration of the local part.

### Concept of the Network Representation Learning Method

Network representation learning method (Zhang et al., 2018) is learning and mastering the node vector in the network. It is a feature learning method, which is the same as learning of an image or text. The code of the assignment group is regarded as the vectors for the network, and it is similar to the node characteristics in the network. The judgment network represents the quality of learning, and the indicators are compared by edge prediction and visual networks. The advantages of node vectors are shown in Table 1.

**TABLE 1:** Advantages of node vectors.

Number	Content
1	In large-scale networks, visual analysis in low-dimensional vector space can clearly observe node relationships and avoid vocabulary gap.
2	Node distance and node performance are defined by calculation. Nodes can be represented by vectors, and data can be processed in the space of vectors.
3	Node vectors can be used as input data for machine learning, avoiding the need to design new machine algorithms.

**FIGURE 2:**
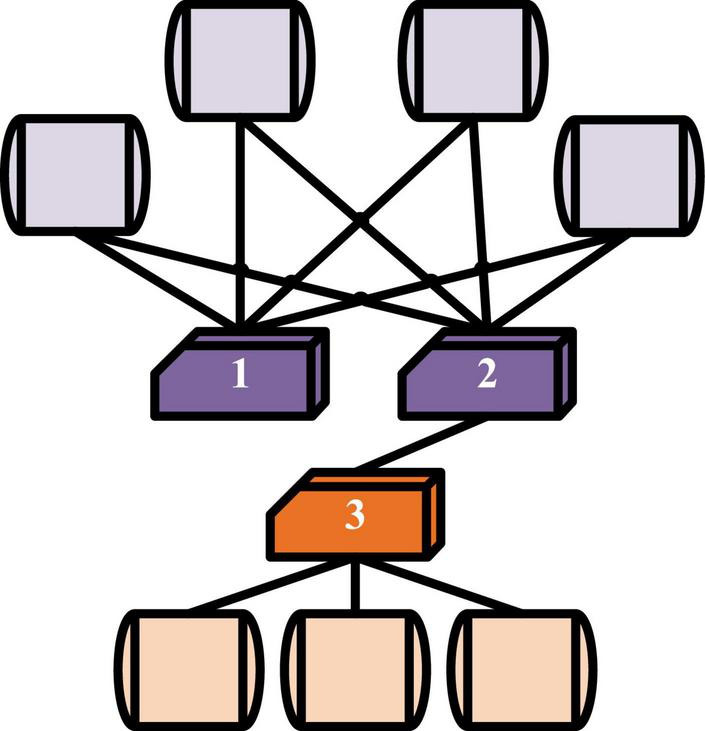
Network proximity.

Social media networks (Üsküplü et al., 2020) are used. If two people have a common interest, they will be more likely to have similar hobbies. In Figure 2, nodes 2 and 3 represent two people, and they are directly connected. However, node 1 and node 2 have a common interest, so are closer they are closer in the low dimension space.

When the adjacent connection matrix of a network model is widely used to describe a network topology, each row vector or column vector can represent one of the nodes, so these vectors can be used to represent the node, and | V| represents the dimensions of the node, namely the total number of all nodes. This symbolic method has the same problem with coding in natural language processing (NLP), that is, it is difficult to choose an appropriate word in another language system, which means that the representation between any two nodes is not completely equivalent. The ultimate goal of NRLM is to use a low-dimensional thought space to describe the whole network. The decomposition algorithm of the matrix is also to learn the low-dimensional ordered space of the original matrix. The purposes of the two are similar, and different algorithms are applied to different matrix decompositions, like Laplacian matrix.

The Laplacian matrix (Belgacem et al., 2021) demonstrates that each data node can be calculated by the adjacent linear weighted combination, and the local weight matrix of each grounding is obtained by the adjacent matrix processing. In the node space, the same weight matrix is used to construct the adjacent points of the node, and the dimensionality reduction process is transformed into the feature decomposition process. The objective function is as follows:


(6) $$\emptyset \,\left(Y\right)=\sum_{i}{\left|{\vec{Y}}_{i}-\sum_{j}W_{i\,j}\,{\vec{Y}}_{j}\right|}^{2}$$

In the above equation, ${\vec{Y}}_{i}$ and ${\vec{Y}}_{j}$ represent the representation vector of the node, and *W*_*ij*_ is the weighted value of X_*j*_. If it is adjacent to node i, the value of *W*_*ij*_ is between 0 and 1. If node i is not adjacent to node j, the value of *W*_*ij*_ is 0, indicating $\sum_{j}W_{i\,j}\,{\vec{Y}}_{j}$ is the weighted reconstruction process of the node.

Laplace feature mapping algorithm (Acosta-Cabronero et al., 2018) aims at the approximate similarity of adjacent nodes in the original space where the adjacent matrix is located in the implicit feature space. The objective function is calculated by:


(7) $$m\,i\,n\,\sum_{i,j}{||y_{i}-y_{j}||}^{2}\,A_{i\,j}$$

*y_i_* and *y_j_* are the representation vectors of nodes i and j in the hidden space, and A is the adjacent matrix of the entire network. After deduction, the following objective function can be obtained by:


(8) $$m\,i\,n\,\sum_{i,j}{||y_{i}-y_{j}||}^{2}\,A_{i\,j}=2\,t\,r\,a\,c\,e\,\left(Y^{T}\,L\,Y\right)$$

In the above equation, L represents the Laplacian matrix of the network.

### Network Structure of Network Representation Learning Model

Network representation learning method is based on the shallow network model, which cannot meet the requirements of the new era. The goal of DL and NRLM is to learn the mapping function and vector in the feature space. Therefore, the two are combined, and NRLM based on DL is constructed.

**FIGURE 3:**
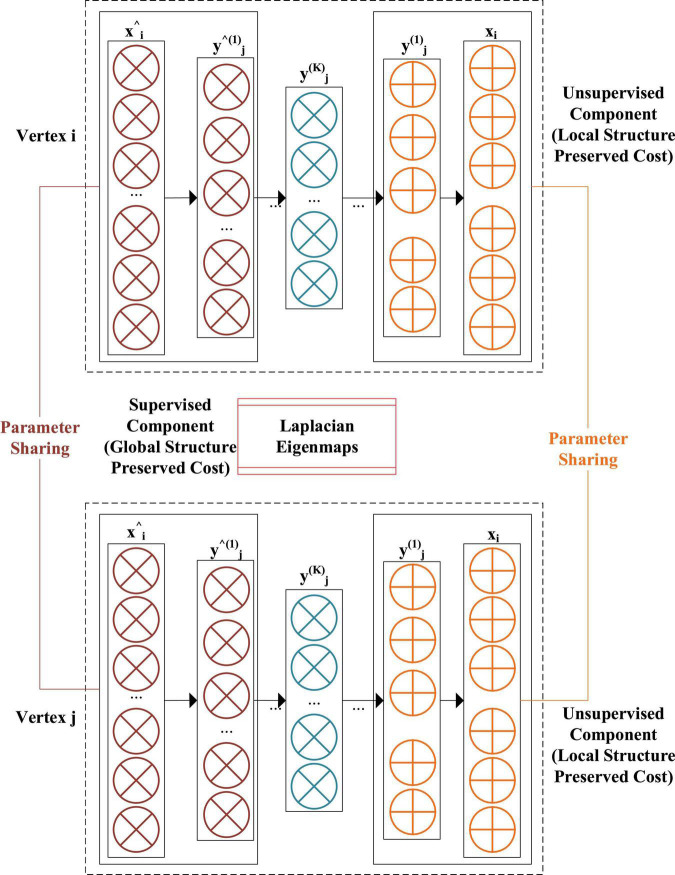
Structure diagram of Structural Deep Network Embedding.

A model called Structural Deep Network Embedding (SDNE) (Wang et al., 2019b) is to obtain high-latitude and nonlinear structural data features in the network to solve the problems in shallow networks. Figure 3 shows the structural schematic of SDNE, which uses SAE to retain adjacent data features of nodes.

After adjacent matrices are input, the adjacent vectors of nodes can be represented by several hidden layers and nonlinear activation functions. The equations for each hidden layer are as follows.


(9) $$y_{i}^{\left(1\right)}=\sigma \,\left(W^{\left(1\right)}\,x_{i}+b^{\left(1\right)}\right)$$

(10) $$y_{i}^{\left(k\right)}=\sigma \,\left(W^{\left(k\right)}\,y_{i}^{k-1}+b^{\left(k\right)}\right),k=2,\ldots ,K$$

In Equations 9, 10, W and b represent the parameters in the hidden layer according to the frame and the bias, and y is the hidden layer. The non-zero value of the input adjacent vector needs to be weighted to reconstruct node ${\hat{x}}_{i}$ of SAE because the network model is sparse. And more penalty weights are given to the value of restoring 1 to 0, and the penalty vector is calculated by:


(11) $$b_{i}={\left\{b_{i\,j}\right\}}_{j=1}^{2}$$

The values in the penalty vector are known, so the objective function of the second proximity is achieved by:


(12) $$L_{2\,n\,d}=\sum_{i=1}^{n}{||\left({\hat{x}}_{i}-x_{i}\right)\,⊙b_{i}||}_{F}^{2}$$

When the objective function is designed, it is necessary to ensure that the implicit vectors in the encoders of the two nodes are closer, so that the topological characteristics of the first proximity are preserved. The designed objective function is as follows:


(13) $$L_{1\,s\,t}=\sum_{i,j=1}^{n}s_{i,j}\,{||\left(y_{i}^{\left(K\right)}-y_{i}^{\left(K\right)}\right)\,⊙b_{i}||}^{2}$$

In the above equation, *s*_*i,j*_ represents that whether the two nodes i and j are connected. If they are connected, the value is 1; otherwise, it is 0. Whenever the first proximity is calculated, the Laplace feature mapping is used, and the objective function is obtained by:


(14) $$L_{1\,s\,t}=2\,t\,r\,\left(Y^{T}\,L\,Y\right)$$

In Equation 14, *tr*() is the matrix trace. The final objective function of the network model is obtained by combining the objective function of the first proximity with the objective Korean of the second. The equation is as follows:


(15) $$L_{m\,i\,x}=L_{1\,s\,t}+\alpha \,L_{2\,n\,d}+\gamma \,L_{r\,e\,g}$$

*L*_*reg*_ is the normalized term of the parameters in the model, and α and γ are the super-parameters, which is the optimal value that needs to be obtained by adjusting the parameters.

### Construction of Network Representation Learning Method Based on Deep Learning

Similarity partition is a basic task to be done in feature computing. Network node partition is realized by using training data, and the similarity between samples are obtained. But the traditional metric learning method (TMLM) just obtains a linear Mahalanobis distance, and it cannot show the nonlinear structure of the sample. In recent years, a kind of depth measurement learning technology is widely used in this field. The objective function is optimized and the measurement space is better divided by establishing a deep neural network (Bianco et al., 2018).

**FIGURE 4:**
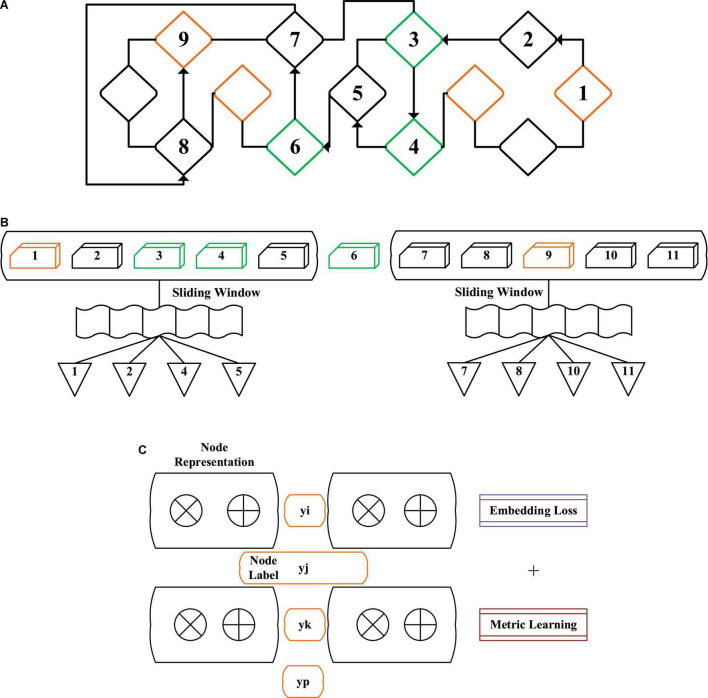
Structure diagram of network representation learning method based on deep learning [**(A)** network random walk link (NRWL), **(B)** generating node sequence link (GNSL), and **(C)** joint training update link (JTUL)].

Therefore, the proposed NRLM based on DL has the following characteristics: (1) the network model can maintain the adjacent relationship between the original network nodes after the representation is embedded; (2) embedding the network model (Shi et al., 2018) can make each node of the same type close to each other, and the nodes of different types can be gradually kept away. As shown in Figure 4, NRLM based on DL has three parts, which are the joint training update link (JTUL), the generating node sequence link (GNSL), and the network random walk link (NRWL). Based on the existing algorithms, the similarity of each node is measured to achieve the goal of mutual proximity of the same type of nodes and mutual distance of different types of nodes.

As for the objective function of the model, if it is known that there are a small number of node labels, the vector initialization distance is shortened by selecting different types of nodes as the initialization nodes and expressing the nodes as vectors before training the model. Whenever different types of nodes are marked, node pairs are built to learn in a specific dimension space.

For each node target vi, the sequence of upper and lower nodes in the sliding window w is w_*v1*_ = v_*i–w*_, v_*i–w*__+__1,…,_ v_*i*__+__*w*_. The model can maximize the probability of node walking sequence and obtain the embedding vector of network nodes by training. The embedding loss function of the model is obtained by:


(16) $$L_{u}=-\sum_{v_{i}\in V}\log p\,\left(v_{i-w},v_{i-w+1},\ldots ,v_{i+w}|v_{i}\right)$$

After the above equations are integrated, the simplified equation is obtained:


(17) $$L_{u}=-\sum_{v_{i}\in V}\sum_{k=-w}^{w}\log p\,\left(v_{i+k}|v_{i}\right)$$

In the above equation,*p*(*v*_*i*+*k*_|*v*_*i*_) is the probability of the upper and lower v_*i*__+__*k*_ nodes in the target which can be observed when target nodes v_*i*_ is known. The calculation equation is as follows:


(18) $$p\left(v_{i+k}|v_{i}\right)=\frac{\text{exp}\in \left(v_{i+k}^{T}\cdot v_{i}\right)}{\sum_{v\in V}\text{exp}\in \left(v^{T}\cdot v_{i}\right)}$$

The gradient algorithm of loss in the above equation uses the construction of the Huffman coding tree (Moffat, 2019) to obtain the updated nodes.

**FIGURE 5:**
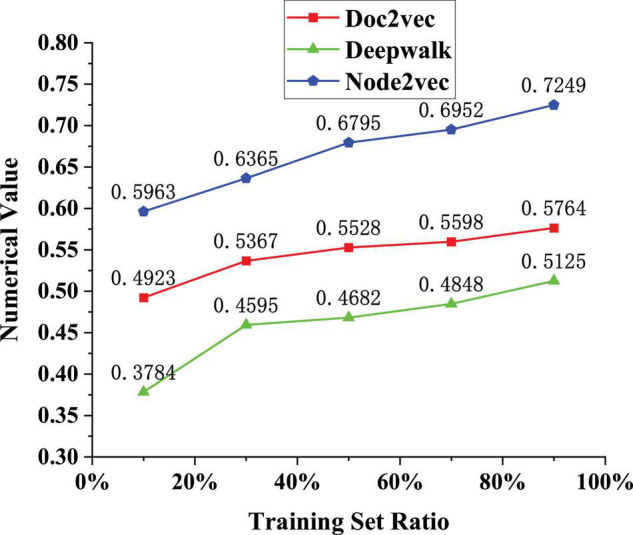
Classification accuracy of different nodes for the labels on the same dataset.

In the process of training the model, k nodes are randomly selected from each type of node to form s. For a pair of different types of nodes in a node v_*i*_ and s, if the two nodes have the same label, their indicator function is 1. Otherwise, it is 0. The metric learning is to maximize the similarity of the same type of nodes and approach each other during training, while the similarity of different types of nodes is minimized and kept away from each other during training. Figure 5 shows the structure diagram of metric learning. In d dimension, different types of nodes have different distances, and three colors represent three different types of node sets.

The whole training process of the model is to minimize the loss function. In the joint training, the random gradient descent method (RGDM) is used to combine the gradient of embedding loss and measuring loss and then reversely transmit them to the network model. For the problem that the computation of all nodes is complex, the negative sampling method (NSM) is used, and the whole training process is shown in Table 2.

**TABLE 2:** Training process of the network model.

Steps	Content
1	Output data: the node sequence w = (w_*v1*_, w_*v2*_,…, w_*vn*_), the label set y and the label set $\hat{y}$ corresponding to the label-free node.
2	Output: the embedding of nodes in network model G is expressed as ε = (e1, e2,…, en).
3	The neural network parameters are initialized, and the label node is represented by e_1_.
4	If the network does not converge or does not reach the maximum iterations, do the followings:
5	Each node in the network v_*i*_ is calculated.
6	v_*i*_ is each node in the starting sequence:
7	According to the loss function, the reverse transport residual Δ is calculated;
8	end for;
9	The equation is updated.
10	end for;
11	end do; (model begins to converge or reaches maximum iterations number of times)
12	Nodes are updated, and ε = (e_1_, e_2_, …, e_*n*_)

### Training Evaluation Indicators

For the evaluation indicators of NRLM, the commonly used methods are the multi-node label classification method (MNLCM) and visualization or prediction link (VOPL). MNLCM is used to evaluate the model. The calculation equation are as follows:


(19) $$p\,r\,e\,c\,i\,s\,i\,o\,n=\frac{T\,P\,\left(A\right)}{T\,P\,\left(A\right)+F\,P\,\left(A\right)}$$

(20) $$r\,e\,c\,a\,l\,l=\frac{T\,P\,\left(A\right)}{T\,P\,\left(A\right)+F\,N\,\left(A\right)}$$

(21) $$F_{1}\,\left(A\right)=\frac{2\,p\,r\,e\,c\,i\,s\,i\,o\,n^{*}\,r\,e\,c\,a\,l\,l}{p\,r\,e\,c\,i\,s\,i\,o\,n\,r\,e\,c\,a\,l\,l}$$

(22) $$M\,a\,c\,r\,o-F_{1}=\frac{\sum_{A\in C}F_{1}\,\left(A\right)}{\left|C\right|}$$

In the above equation, TP (A) is the positive classification case for result A. FP (A) is the negative classification case, and its result is not A1. c is the aggregation of all label nodes, *F*_1_(*A*) is value *F_1_* of label A after calculation. *Macro-F_1_* is the evaluation indicator that treats the weight of various label nodes equally and can comprehensively evaluate the performance of hard node labels.

## Results

### Classification Accuracy of Different Nodes for Labels on the Same Dataset

The performance of the network model needs to be tested. The same kind of datasets is used and the classification accuracy of different nodes on labels is used to judge the applicable performance of the model. The datasets are Doc2vec (node vector representation), Deepwalk (network structure information), and Node2vec (breadth search strategy). The Doc2vec method is an unsupervised algorithm that learns fixed-length feature representations from variable-length text such as sentences, paragraphs, or documents. It can obtain the vector representation of sentences, paragraphs, and documents and is an extension of Word2Vec. It has some advantages, such as not fixing the sentence length and accepting sentences of different lengths as training samples. Deepak is to solve the problem of node embedding in the graph. Graph Embedding technology expresses the nodes in the graph in the form of low-dimensional dense vectors and requires that the similar nodes in the original graph (different methods have different definitions of similarity) are also close in the low-dimensional expression space. Expression vectors can be used for downstream tasks such as node classification, link prediction, etc. Node2vec is a model used to generate node vectors in the network, and the input is the network structure (which can be weightless). The output is the vector of each node. The main idea is to directly guide the word2vec package, sample through a specific walking method, and generate a corresponding sequence for each point. These sequences are treated as text and imported into the bow or skip-gram model in word2vec, and the vector of each node (corresponding to the vector of each word in word2vec) can be obtained. The test results are shown in Figure 5.

The analysis of the data results in Figure 5 shows that with the increase of the number of training sets (10→90%), the classification accuracy of the model for three node labels is improved, and the accuracy of Doc2vec is 0.7249 when the training set is 90%. The accuracy of Deepwalk is 0.5764 when the training set is 90%; the accuracy of Node2vec is 0.5125 when the training set is 90%. Therefore, it is believed that this network representation learning algorithm can make full use of the existing label information to embed network nodes. The final generated vector has better classification effect. The application of the algorithm in practical work can also reflect the role of label nodes. Whether user classification is necessary depends on whether it is always beneficial to user research goals. In many cases, placed in a specific task scenario, the user’s behavior will not show essential differences in the face of a specific product. It is necessary to understand how “behavior” arises to understand this problem. Any kind of human behavior is the result of the combined action of dozens of internal and external factors. In different scenarios, although there are still so many types of influencing factors, the influence weight of each factor will change. In the process of user interaction with a specific product, the constraints formed by the product’s own characteristics may become the most important factor affecting user behavior, rather than some characteristics of the user itself, such as gender, age, or personality. Since the characteristics of a particular product are unique, people with different intrinsic characteristics may behave similarly to the same product. At this point, the value of user research lies in observing and discovering how users react to these restrictive factors of the product rather than studying how to classify people.

### Classification Effect of the Same Dataset in Different Parameter Weights

**FIGURE 6:**
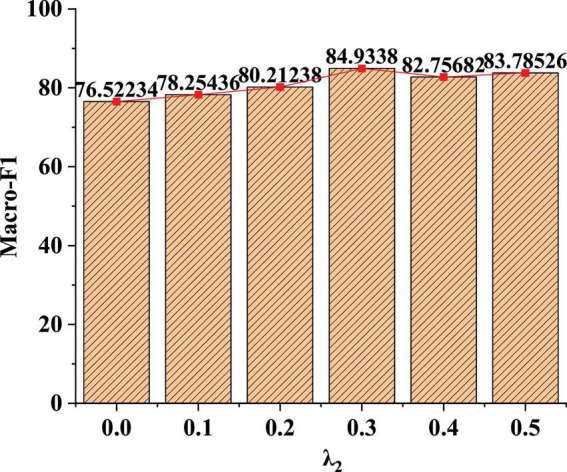
Recognition effect of model Macro-F1 value under different parameter weights.

The dataset used is the Flickr social network dataset. This dataset contains social network relationship data scraped by websites. In this dataset, each node is a user in Flickr, and each edge is a friendly relationship between users. Additionally, each node has a label that identifies the user’s interest group. The model evaluation indicators in part 3.5 are used to judge the effect of the model to compare the classification effects of the same dataset under different parameter weights λ_2_. The specific test results are shown in Figure 6.

Figure 6 shows that when the value of λ_2_ is 0, the learning loss of non-fusion measure is shown. If λ_2_ is 0.3, the node classification effect of the model is the highest. When λ_2_ is small, this learning method has no obvious effect on the classification. When λ_2_ is 0.4, the weight is too high in the classification process, which affects the classification effect of adjacent labels. Therefore, parameter weight λ_2_ is recommended to be set as 0.3.

**FIGURE 7:**
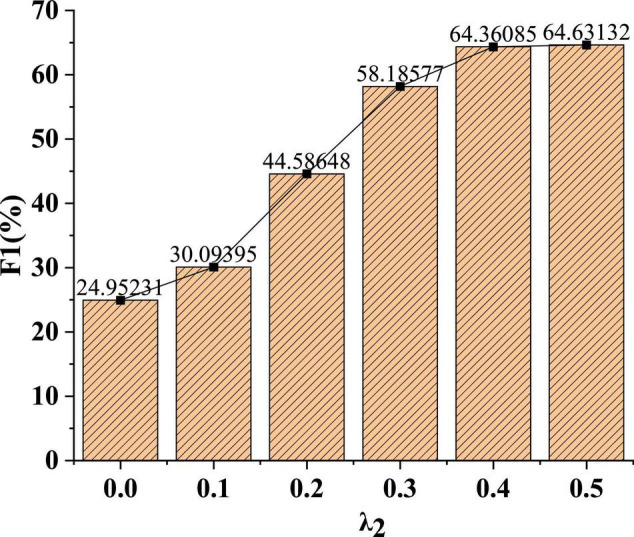
Recognition effect of model F1 value under different parameter weights.

In Figure 7, as the parameter weight increases, the recognition effect of the model F1 value is gradually enhanced. When the parameter weight λ_2_ is 0.1, the F1 value of the model is 30%. When the parameter weight λ_2_ is 0.4, the F1 value of the model is 64.4%. After that, the F1 value of the model can stabilize at around 64%. The data show that the proposed model can achieve stability under certain parameter weights.

**FIGURE 8:**
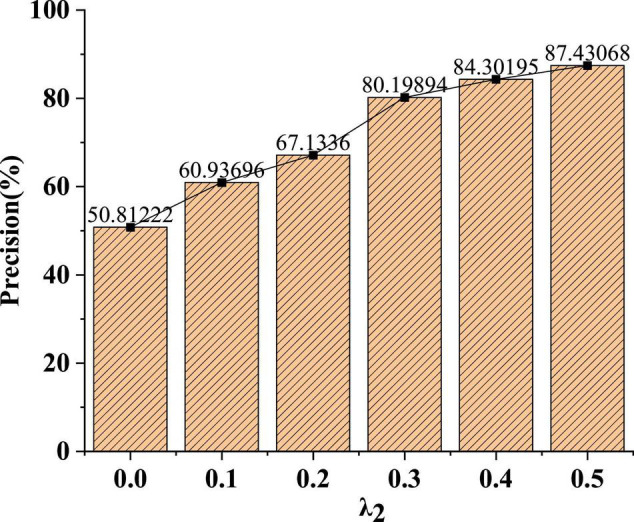
Change curve of model recognition accuracy under different parameter weights.

In Figure 8, under different parameter weights, the recognition accuracy of the model will gradually increase. When the parameter weight λ_2_ is 0.1, the recognition accuracy of the model is only 60%. In addition, after the parameter weight λ_2_ is increased to 0.3, the average recognition accuracy of the model can reach about 84%.

**FIGURE 9:**
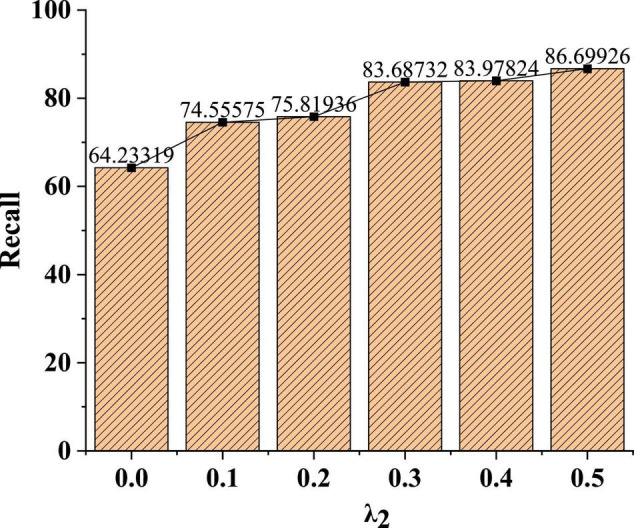
Recognition effect of model recall under different parameter weights.

In Figure 9, the larger the parameter weight, the better the recognition recall of the model. When the parameter weight λ_2_ is 0.1, the recognition recall rate of the model is only 74%. With the increase of the parameter weight, after the parameter weight λ_2_ increases to 0.3, the average recognition recall rate of the model can reach more than 83%.

Two orientations are used to select the appropriate dimension:

One is insight orientation. This refers to the key dimensions that the researcher finds that affect user behavior based on experience and thinking accumulated in the target product and product field, as well as through qualitative research methods such as observation and interviews to understand users. This method is strong in-depth, and the dimension discovered may be more striking to the essence.

The other is statistical orientation. That is, through quantitative research methods such as questionnaires and log analysis, the key factors affecting user behavior are analyzed in quantitative data. These methods are more scientific and more convincing. But the discovered dimension may only be an intermediate factor, not necessarily an essential factor.

In practice, the statistical approach is more demanding on organizational resources, while the insight approach is more efficient. Therefore, when the resources are sufficient, the insight method and the statistical method are used in combination, and the most likely candidate dimensions are first screened out by the insight method and then verified by the statistical method. When resources are limited, it is recommended to use only the insight method. Of course, whether the insight method works depends on the researcher’s own skills, which not only refers to the mastery of the ideas and methods of user research itself but also refers to the depth of understanding of the target product and its field.

### User Identification Test of Network Representation Learning Method

**FIGURE 10:**
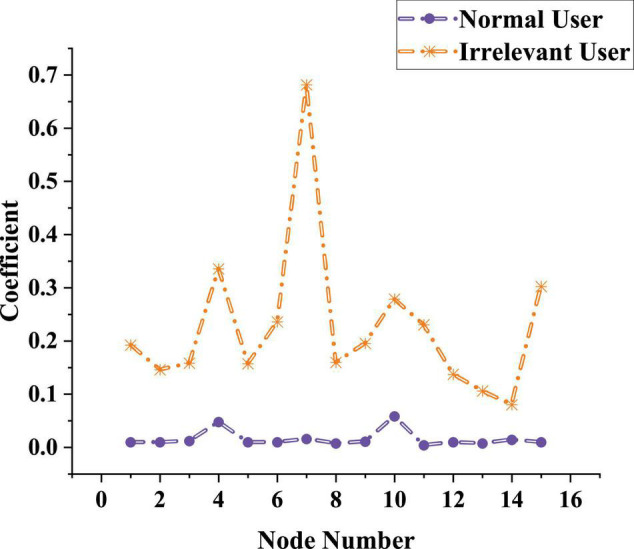
Distinguishing ability of network representation learning method for users’ types.

After the parameters suitable for the model are obtained, NRLM is used to identify the user’s types in the network learning process, and judges whether the model can be used in the actual learning process by identifying the user types. The test results are shown in Figure 10.

The data Figure 10 show that the red curve represents the correlation indicator of related users when using the network, and the blue curve shows the correlation indicator of unrelated users when using the network. The red curve is kept below 0.1. Although the lowest value of the red curve is lower than 0.1, it is maintained at 0.2–0.3. There is no overlap between the two curves, indicating that the model can effectively distinguish the two users, and can also accurately list the correlation indicator of users. Therefore, NRLM can be applied to students’ online education.

## Discussion

This study proposes a network representation learning algorithm based on a structured deep network embedding model with the help of AE under DL. The concept and structure of the model are elaborated. Its performance is tested. Node labels and datasets are trained and found that with the increase in the number of training sets, the model’s classification accuracy for the three-node labels improved. The accuracy of Doc2vec is 0.7249 when the training set is 90%. When the training set is 90%, the accuracy of Deepwalk is 0.5764. When the training set is 90%, the accuracy of Node2vec is 0.5125. Therefore, the proposed network representation learning algorithm can fully utilize the existing label information to embed network nodes. The final generated vector has better classification performance. The application of the algorithm in practical work can also reflect the role of label nodes. In different scenarios, although there are still many types of influencing factors, the influence weight of each factor will change. In the process of user interaction with a specific product, the constraints formed by the product’s own characteristics may become the most important factor affecting user behavior, rather than some characteristics of the user itself, such as gender, age, or personality. Since the characteristics of a particular product are unique, people with different intrinsic characteristics may behave similarly to the same product. The proposed model can effectively distinguish two users and can also accurately list the relevant indicators of users. Therefore, NRLM is applied to students’ online education, which is an innovative teaching strategy and provides a theoretical basis for improving teaching methods. The disadvantage is that the research on the model is not very deep. In future research, factors such as proximity and structural features will be added to test the model’s performance, and different images, audio, and video will be used to study the recognition ability of the model and optimize it.

## Conclusion

Online learning based on networks is proved to be an important way for modern people to live and work. Machine learning provides important support in online learning. As a new learning method, DL plays a huge role in NLP, analyzing media images and recommendation systems. The study aims to improve the learning effect of students by separating irrelevant users from the people who need learning resources and using AE in DL. Combined with the online learning network model, NRLM is proposed. After the establishment of the model, the parameters of the model are analyzed. The applicable parameter λ_2_ of the model is 0.3 by using the dataset and label nodes. It is also proved that the model can effectively distinguish relevant users and unrelated users and list the relevant indicators. It is believed that NRLM can guarantee users’ learning effect in students’ online education. Innovative teaching strategies provide a theoretical basis for improving teaching methods. The disadvantage is that the research on the model is not very deep. In future studies, factors such as proximity and structural characteristics will be added to test the performance of the model. Different images, audio, and video are used to study the recognition ability of the model and optimize it.

## Data Availability Statement

The raw data supporting the conclusions of this article will be made available by the authors, without undue reservation.

## Ethics Statement

The studies involving human participants were reviewed and approved by the Zhoukou Normal University Ethics Committee. The patients/participants provided their written informed consent to participate in this study. Written informed consent was obtained from the individual(s) for the publication of any potentially identifiable images or data included in this article.

## Author Contributions

All authors listed have made a substantial, direct, and intellectual contribution to the work, and approved it for publication.

## Conflict of Interest

The authors declare that the research was conducted in the absence of any commercial or financial relationships that could be construed as a potential conflict of interest.

## Publisher’s Note

All claims expressed in this article are solely those of the authors and do not necessarily represent those of their affiliated organizations, or those of the publisher, the editors and the reviewers. Any product that may be evaluated in this article, or claim that may be made by its manufacturer, is not guaranteed or endorsed by the publisher.
